# Evaluation of Preference of Pain Scale in Children using Novel Animated Emoji Scale in Nepal: An Observational Study

**DOI:** 10.31729/jnma.9140

**Published:** 2025-07-31

**Authors:** Ankita Agrawal, Manisha Upadhyay

**Affiliations:** 1 Department of Pedodontics and Preventive Dentistry, Universal College of Medical Sciences, College of Dental Surgery, Bhairahawa, Lumbini, Nepal

**Keywords:** *animated emoji scale*, *pain assessment tool*, *visual analogue scale*, *wong-baker faces pain rating scale*

## Abstract

**Introduction:**

Pain is a common concern for pediatrics patients, maybe challenging because the child cannot properly explain the extent of pain they are experiencing. Several pain assessment tools have been developed and introduced in dentistry that facilitate self-report of pain in children. Three such pain rating scales Animated Emoji Scale, Visual Analogue Scale and Wong-Baker Faces Pain Rating Scale were used in this study to evaluate the pain perception among pediatric patients. This study was aimed to evaluate the preference of pain scale among these three scales in pediatrics patients.

**Methods:**

This hospital based cross sectional study was carried out from January till June in 182 children aged between 3-14 years divided into three groups on the basis of age: Group A (3-6 years, Group B 7-10 years, and Group C 11-14 years). Ethical clearance was taken (Reference number: UCMS/IRC/02/25). Pain score was recorded among these children using Animated Emoji Scale (AES), Visual Analogue Scale (VAS) and Wong-Baker Faces Pain Rating Scale (WBFPS). The data were entered into Excel and analyzed using IBM SPSS Statistics for Windows, version 26 (IBM Corp., Armonk, N.Y., USA).

**Results:**

Spearman’s rank correlation coefficient evaluated a positive correlation among the three pain rating scales. Chi-square test showed statistically significant association between age group and preferred pain scale (p<0.001).

**Conclusions:**

AES was most preferred in younger age groups from age 3-10 years whereas elder children 11-14 years relied more on VAS as a self-reporting tool.

## INTRODUCTION

During a dental procedure, pain is a common concern for patients, parents and pediatric dentists. Understanding the causes and management of pain in children is crucial for effective dental care.^1^ While adults can easily explain their pain, it becomes challenging among pediatric patients.^2^ Children can represent any unpleasant experience induced by a conditioned stimuli such as site of a syringe, airotor, sharp pointed instruments or sound of suction as pain.^3^ Pain perception in children causes several changes in their emotional states like fear, anxiety and sadness. These emotional factors occur not only in response to pain but also with any unwanted site or unpleasant stimuli that trigger undesired behavior in patients.^4,5^ Several pain assessment tools have been developed and introduced in dentistry that facilitate self-report of pain in children. We used AES to offer a scale that is engaging, intuitive and kid’s friendly. The present study compares the preference of three such pain rating scales, Animated Emoji Scale (AES), Visual Analogue Scale (VAS) and Wong-Baker Faces Pain Rating Scale (WBFPS) in children attending pediatric dental outpatient department.

## METHODS

This was an obsevational cross-sectional study carried out in 182 children attending the Department of Pedodontics and Preventive Dentistry of a tertiary care center in Nepal from January 2025 to June 2025 for a period of 6 complete months. Children in age group 3-14 years, attending Pediatric dental OPD for the first time and willing for treatment were enrolled in the study. Ethical clearance was taken from the institutional review committee (Reference number: UCMS/IRC/02/25). Informed consent and assent were obtained from parents and children before enrollment in the study. Confidentiality of the recorded data was maintained. The sample selection was done using a non-probability convenience sampling method and divided into three groups on the basis of age: Group A: 3-6 years, Group B: 7-10 years, and Group C: 11-14 years. Children with mental disabilities, speech impairments, hearing and visual difficulties and parents who declined to participate were excluded from the study.

The sample size was estimated using G*Power software (version 3.1.9.2) for a one-way ANOVA (fixed effects, omnibus, one-way) to compare the mean pain scores across three age groups (3-6 years, 7-10 years, and 11-14 years) for each pain assessment tool (VAS, WBFPS, AES). Based on data from previous studies,^10,15^ the following parameters were used:

Effect size (f) = 0.31

Significance level (α) = 0.05

Power (1-β) = 0.95

Number of groups = 3 (age groups)

The calculated sample size was 165 participants. To account for incomplete responses, a 10% buffer was added, bringing the total to 182 participants.

In addition to comparing mean pain scores across age groups using one-way ANOVA, this study also evaluated the association between pain scores measured using VAS, WBFPS, and AES by applying Spearman’s rank correlation coefficient (p) after the normality testing. The minimum required sample size to detect a statistically significant Spearman’s correlation of p = 0.30 at 5% level of significance and 95% power of statistical test 138 participants was required.

Since the study enrolled 182 participants, this sample size was sufficient to power both the ANOVA and correlation analyses used in this study.

Children with first dental visit and requiring any type of dental treatment such as extraction, endodontic therapy (pulpectomy, pulpotomy, root canal treatment, indirect pulp capping, direct pulp capping). Each patient’s dental pain was measured using three different scales: VAS, WBFPS and AES. Each scale was presented to the child in the sequence from VAS to WBFPS to AES; all the scales were presented to each child by a single investigator, and pain scores were immediately recorded to ensure reliability and avoid bias.

Visual Analogue Scale is a self-reporting number scale that measures the characteristic or attitude of pain. It is a simple tool consisting of a 0 to 10-cm line on another end, 0 showing no pain, 5 mild pain and 10 showing the worst pain. The scale is measured in millimeters from the left-hand end of the line to the point that the child marks. After the procedure, children were asked to point out the number showing on the line representing the level of perceived pain intensity by them.^6^

Wong-Baker Faces Pain Rating Scale is commonly used in pediatric dental and medical settings specifically with younger children. It represents six faces showing various feelings ranging from “no hurt to hurts worst (most positive to most negative feelings)”. After completion of procedure the children were asked to select the face that best expressed how they felt.^7,8^

Animated Emoji Scale is a novel tool to assess the intensity of pain. This tool uses emoji representing facial expressions which represents various emotions, to which children relate and express themselves despite their verbal insufficiency. This scale consists of six animated emoji faces representing various expressions of faces ranging from happy to crying. After completion of the procedure children were given a colored laminated chart and were asked to select one of the best related pictures to their feelings.^9,10^

The pain scores were recorded in all the participants using each scale (VAS, WBFPS and AES). The data collected were tabulated and subjected to statistical analyses using IBM SPSS Statistics for Windows, version 26 (IBM Corp., Armonk, N.Y., USA). Descriptive analysis of all the explanatory and outcome parameters was performed using frequency and proportions for categorical variables, and using mean and standard deviation for continuous variables. Frequency and the mean difference in pain scale between different age groups were calculated by ANOVA test. The correlation between AES, VAS and WBFPS at different age groups was calculated by using Spearman’s rank correlation coefficient (ρ).

## RESULTS

A total of 182 children were included in the final analysis who visited the pediatric dental department for the first time and were ready for the procedure. Among them, 99 (54.40%) were males and 83 (45.60%) were females. The male-to-female ratio was 1.19:1.00. All children were divided into three groups based on age: Group A (3-6 years) with 83 (45.60%) children, Group B (7-10 years) with 75 (41.21%) children, and Group C (11-14 years) with 24 (13.19%) children (Table 1).

**Table 1:** Frequency Distribution of Children by Different Age Groups (n=182)

Groups (Age in years)	n (%)
Group A (3-6)	83(45.60)
Group B (7-10)	75(41.21)
Group C (11-14)	24(13.19)

**Table 2:** Comparison of pain scores rated by pain scales in all three age groups (n=182).

Scale	Group A	Group B	Group C
Visual Analogue Scale	4.41±2.90	4.24±2.74	2.54±2.86
Wong-Baker Faces Pain Scale	4.53±3.19	4.78±2.22	3.42±3.61
Animated Emoji Scale	4.36±3.31	4.76±2.60	3.42±3.56

**Table 3:** Correlations between pain scores rated by pain scales in all three age groups (n=182).

	Group A	Group B	Group C
	Correlation Coefficient(r)	Correlation Coefficient(r)	Correlation Coefficient(r)
AES Vs VAS	0.784	0.618	0.724
AES Vs WBFPS	0.930	0.840	0.973
VAS Vs WBFPS	0.761	0.505	0.748

AES = Animated Emoji Scale ; VAS = Visual Analogue Scale ; WBFPS = Wong-Baker Faces Pain Scale

The mean pain scores recorded using the Visual Analogue Scale were 4.41 ± 2.90 in Group A, 4.24 ± 2.74 in Group B, and 2.54 ± 2.86 in Group C. The Wong-Baker Faces Pain Scale showed scores of 4.53 ± 3.19 for Group A, 4.78 ± 2.22 for Group B, and 3.42 ± 3.61 for Group C. Similarly, the Animated Emoji Scale recorded scores of 4.36 ± 3.31 in Group A, 4.76 ± 2.60 in Group B, and 3.42 ± 3.56 in Group C (Table 2).

The correlation between AES, VAS, and WBFPS across different age groups was calculated using Spearman’s rank correlation coefficient (ρ). The correlation values were as follows: AES and VAS: 0.784, 0.618, 0.724; AES and WBFPS: 0.930, 0.840, 0.973; VAS and WBFPS: 0.761, 0.505, 0.748 (Table 3).

The frequency distribution of preferred pain scales among the children was calculated using the Chi-square test. AES was preferred by 118 (64.84%) children, VAS by 41 (22.53%), and WBFPS by 23 (12.64%).

The association between age group and preferred pain scale was calculated using the Chi-square test. The results showed a statistically significant association between age group and preferred pain scale (p < 0.001). In Group A, 65 (78.31%) children preferred AES. In Group B, 45 (60.00%) children preferred AES. In Group C, 13 (54.17%) children preferred VAS (Table 4).

**Table 4:** Association between age group and Preferred pain scale (n=182).

Age group	Pain Scale			P Value
	AES	VAS	WBFPS	
Group A	65 (78.33)	7 (08.44)	11 (13.33)	<0.001
Group B	45 (60)	21 (28)	9 (12)	
Group C	8 (33.33)	13 (54.17)	3 (12.50)	

AES=Animated Emoji Scale ; VAS= Visual Analogue Scale ; WBFPS= Wong-Baker Faces Pain Scale

## DISCUSSION

Proper understanding of the feelings and behaviour of the child and the exact reasons for the fear and anxiousness, can help in managing the pain efficiently. This could be achieved by thorough knowledge and understanding of the psychology behind the particular behaviour of the child.^11^ In early stages of life, children could not express their feelings due to their limited verbal communication but with age they develop a broader vocabulary and the better ability to communicate what they feel.^12,13^ Conventionally, VAS and WBFPS are being frequently used to assess the pain in children as a self-report tool.^6^ In this study of 182 patients we compared AES with frequently used VAS and WBFPS, and it was found that AES was most preferred pain assessment scale compared to these conventional self-reporting pain assessment scales in the children aged 3-10 years whereas VAS was the scale of choice among elder children aged 11-14 years. These findings were consistent with various studies.^14^ Since children of younger age groups are more inclined towards colourful pictures of different cartoon characters and in recent time due to introduction and increased exposure of mobile phone into the younger kid’s life, they all felt connected to colourful emojis showing different expressions and they were well versed with different expression of emojis and their meanings. Out of all the three pain rating scales AES was the most colourful one with familiar character for the younger kids therefore, they tend to prefer it more over the other two scales.

In response to unpleasant stimuli, cognitive development begins early in childhood, which can also be inferred by our study where there is a decrease in the mean pain score by children with increasing age. The mean pain score for VAS in our study was similar to the mean pain score of (novel animated emoji scale) in the study by Khatri et al.^10^ and Prasad et al.^15^ Whereas the mean pain score of WBFPS and AES was showing a different pattern than VAS. The mean pain score for these two scales (WBFPS and AES) was lowest score in eldest age group and highest in middle age group which was in contrast with Khatri et al.^10^

Liking preference of the scale depends on the individual interest of the patients, and also on the understanding of the children to interpret their feeling of pain they are undergoing.^16^ It was observed that VAS scale was most difficult to explain to younger children as it is a in a linear pattern and children aged 3-6 years express difficulty in understanding the pattern and it take more time to explain the same, which was comparatively easier for the WBFPS and there was no difficulty observed in recording the AES by these children at all. Hence, it may be assumed that due to more familiarity of the children with emojis themselves, younger groups have chosen AES over others. Children do not have the same cognitive abilities as adults to quantify and qualify abstract phenomena, and younger children may not cognitively be able to understand and use self-reporting measures of pain like VAS which looks comparatively unfamiliar and difficult from AES and WBFPS.^17^ In support of this theory, we found that children who were able to understand the concept of the VAS and preferred it over the other two scales were significantly older than those who did not understand, 11-14 year old were more likely to understand the VAS than 3-10 year old. The result of this study suggested that pain experience was easily explained by AES in the younger group and VAS in the eldest age group. Further, more studies with larger samples should be conducted to promote our results.

## CONCLUSIONS

Self-reporting tool for pain assessment are valuable asset in pediatric dentistry as it enables the child to express their pain experience and helps the dentist to understand the child better. AES was most preferred in younger age groups from age 3-10 years whereas elder children 11-14 years relied more on VAS as a self-reporting tool.

## Data Availability

The data are available from the corresponding author upon reasonable request.
